# Barriers and Coping Strategies to Assistive Device Use Among Individuals With Physical and Hearing Disabilities in the Ashanti Region of Ghana: A Qualitative Study

**DOI:** 10.1002/hsr2.73039

**Published:** 2026-08-12

**Authors:** Jeffrey Kwame Owusu Ansah, Enoch Acheampong, Ros Yaw Owusu‐Ansah, Grace Kisiwaa Agyei, Vida Kasore, Justice Owusu Agyemang, Betty Agyei Kponyo, Jerry John Kponyo, Elizabeth Owusu Ansah

**Affiliations:** ^1^ Kwame Nkrumah University of Science and Technology Kumasi Ghana

**Keywords:** Ashanti Region, assistive device, barriers and coping strategies, hearing disability, physical disability

## Abstract

**Background and Aims:**

It is estimated that globally, about 1.3 billion people live with disabilities, with 80% residing in low‐ and middle‐income countries. In Ghana, individuals with physical and hearing impairments face barriers accessing assistive devices. *This study explored the barriers and coping strategies of assistive device use among individuals with physical and hearing disabilities in the Ashanti Region of Ghana*.

**Methods:**

The study used a qualitative approach with a phenomenological design conducted among 21 individuals with hearing and physical impairment from five districts in the Ashanti Region. The participants were selected using a purposive sampling technique. Face‐to‐face interviews were conducted using an interview guide.

**Results:**

The key barriers to the use of assistive devices among people with hearing and physical impairments included high cost, limited availability, and stigma. Coping strategies that emerged from the findings were adaptation through practice, mental resilience of the users, and the external support they had from family and friends who provided financial assistance or some of the assistive devices.

**Conclusion:**

In Ghana's Ashanti Region, assistive devices play a crucial role in the lives of people with disabilities, yet stigma, high cost, and limited availability continue to be major barriers to their usage. The government should launch subsidy programs to increase accessibility and affordability in order to address these issues. Furthermore, focused community awareness initiatives should be started in order to reduce stigma and change public perceptions of people with disabilities, allowing them to fully engage in society.

## 1 Introduction

*The aging population and growing number of cases of non‐communicable diseases are contributing factors to the increased prevalence of disability worldwide* [1]. In low‐ and middle‐income countries, the challenge of disability is compounded by systemic barriers to accessing assistive technologies, which are critical tools for enabling independence and social participation. Many people with impairments rely on assistive technology services to carry out their daily tasks. The difficulties that people with impairments encounter on a daily basis may be greatly reduced by assistive technology. Without the use of these technologies, people with impairments would not have access to opportunities [2]. Assistive technologies are increasingly important in rehabilitation to enhance functionality [3]. The role of assistive technology as a means and an end in the realization of the rights of individuals with disabilities is acknowledged in the global report on assistive technology [4]. However, there are obstacles to using these devices, such as the lived experiences of these people are not considered during the manufacturing of these assistive devices, as decision‐making is made by the manufacturer rather than considering the user's needs [5].

In many parts of Africa, getting access to assistive devices and rehabilitation services remains severely limited due to high costs, over‐reliance on imports, and a shortage of personnel and weak governance [6]. A study conducted in four sub‐Saharan African countries, namely Namibia, Zambia, Malawi, and Zimbabwe, concluded with having a high procurement challenge of assistive devices, where 49.7%, 20%, 42.7%, and 38.9% of people with impairments who required assistive technology did not own it [7]. The high cost of assistive devices resulted in 44% of people with disability in Botswana and 67% of people in Swaziland who needed assistive devices on the continent not receiving it, according to a study by Olarewaju et al. [8]. According to a report by Mahmoudi‐Dehaki [9], assistive technology has the potential to improve the lives of individuals with disabilities; however, in many places, these gadgets are either unavailable or too costly. This lack of access makes it difficult for persons with disabilities to become independent, which makes it impossible for them to integrate into society, thereby causing disadvantages to them. Cultural and societal obstacles greatly influence the adoption and utilization of assistive devices, hindering individuals. Numerous individuals with disabilities encounter stigma within their communities, while others experience discrimination or social exclusion.

In Ghana, Acheampong et al. [10] associate barriers faced by individuals with disabilities with societal stigma, limited awareness of available assistive devices, and inadequate financial assistance. For an assistive technology to be used and accepted, particularly by the hearing impaired, it has to be shaped by socio‐cultural contexts, social norms, and users' lived experiences within their environments; this influences the technology being adopted, adapted, or avoided by the person with disability. A study by Acheampong et. al. [10] stated that 28.8% of participants did not use assistive devices because of the stigma associated with their use. Because assistive gadgets can draw attention to disability, some people have avoided using them out of fear of stigma or social reactions. According to [11], persons with disabilities experience prejudice and social stigma, and this leads to social exclusion, which affects their participation in society and their lived experiences. These can also affect their decision to use an assistive device.

Individuals with hearing impairments and persons with other forms of disabilities have coping strategies which they employ to manage the stigma around them and also the environmental barriers which they face daily [12]. According to Osam et al. [13], families are essential for obtaining assistive technology since they frequently band together to pool resources for essential gadgets. This reliance on family support is indicative of larger social trends in Ghana, where unofficial networks address shortcomings in the availability of official assistive technology. A key component of problem‐focused coping is being proactive in addressing obstacles. For access to assistive technology and related services, people in the Ashanti Region frequently rely on community‐based rehabilitation initiatives. Through the provision of essential resources and community support, these initiatives seek to improve the quality of life for people with disabilities [14]. Participating in group activities and support groups helps people with physical and hearing impairments overcome these obstacles by reducing feelings of loneliness and fostering a sense of belonging [15]. Despite the significance of assistive technology for people with hearing and physical impairments, little is known about the particular obstacles to using and gaining access to these devices and the coping strategies adopted in the Ashanti Region of Ghana. The need for this research is highlighted by the fact that this information gap impedes the creation of efficient treatments and support services catered to their needs. There are many unanswered questions about the unique lived experiences of people with both physical and hearing impairments in the same study, despite the growing body of research on assistive technology access in sub‐Saharan Africa, especially in Ghana. Few studies have looked at the interaction between barriers and coping mechanisms simultaneously in an urban setting in a low‐ and middle‐income country. Most of the existing studies have focused on either barriers or a specific type of disability. Besides this, there is still little research that examines similar dynamics in the Ashanti Region, one of the most populous areas in Ghana. The originality of this study lies in three areas. First, it is one of the few studies in the Ghanaian context to simultaneously study both physical and hearing disability groups in the same qualitative study, thus providing for cross‐disability comparison of barriers and coping strategies. Second, it adds to the small body of phenomenological research on assistive technology use in West Africa, where most research has used quantitative survey methods. Third, it offers empirically grounded policy insights specific to the Ashanti Region, a context that has received insufficient attention in the international disability and assistive technology literature. *Therefore, this study aimed to explore the barriers to assistive device use and the coping strategies employed by individuals with physical and hearing disabilities in the Ashanti Region of Ghana*.

## 2 Methods

### 2.1 Study Design and Setting

*This study employed a qualitative research approach, specifically a phenomenological design, to explore the lived experiences of individuals with physical and hearing disabilities in the Ashanti Region of Ghana. The phenomenological design was chosen to capture the depth of participants' personal experiences with assistive device use, as the majority of existing research has employed quantitative methods focusing solely on either coping mechanisms or barriers to assistive technology use*. The Ashanti Region has its capital in Kumasi, and it is in the southern part of Ghana. The region has both urban and rural districts, and this gives diverse socio‐economic conditions. *According to the* Ghana Statistical Service [16], *the Ashanti Region has a total of 10,385 persons with physical impairment and 4501 persons with hearing impairment, providing the broader population context for this study*. Although the Ashanti Region has its urban areas having better access to healthcare, there are still challenges in its infrastructure, and its socio‐cultural way of living affects access to assistive technologies by persons with disabilities. The Ghana Statistical Service [16] reports that there are more disabled individuals in urban areas than in rural ones. Because of this, pre‐urban and urban districts were selected. This offers a diverse socioeconomic background relevant to research on assistive devices. The viewpoints of the study participants were gathered using a qualitative study approach.

### 2.2 Sampling

People with hearing and physical impairments who lived in Ghana's Ashanti Region made up the study population. Five urban and pre‐urban districts, Ejisu, Oforikrom, Asokwa, Kumasi Metropolitan, and Asokore Mampong, were purposely selected from the 43 districts that make up the Ashanti Region. These districts were chosen based on three criteria: (1) their urban and pre‐urban character, which reflects a higher concentration of people with disabilities as reported by the Ghana Statistical Service [16], (2) their accessibility to the research team, which made it possible to record a wider range of lived experiences, and (3) their varied socioeconomic circumstances, which made it possible to record a wider variety of lived experiences. Because the study's focus was on urban and pre‐urban settings, where assistive device services and organizations for people with disabilities are more likely to be present, rural areas were excluded.

The Department of Social Welfare and recognized organizations for people with disabilities were used to identify participants within each district that was chosen. The research team got in touch with these organizations and asked for recommendations of people who fit the inclusion criteria. To guarantee diversity in age, gender, kind of disability, and educational background, participants were purposely selected from individuals who were referred. Instead of focusing on a small or homogeneous group, this method made sure that the sample represented the variety of experiences among the research population.

### 2.3 Data Collection

The study used a semi‐structured interview guide, an audiotape, and a jotter for data collection through an in‐depth face‐to‐face interview. The interview lasted 45–60 min and was audio recorded with the participants' informed agreement. During and shortly after each interview, the interviewer took field notes to document contextual observations and nonverbal clues that could improve the analysis. There were no non‐participants present during the interview sessions to ensure confidentiality.

### 2.4 Study Population

*Inclusion criteria:* Individuals with physical or hearing impairment, aged 18 years and above, and registered members of organisations for persons with disabilities in the selected districts. Adults aged 18 and above were included as this is the legally recognised age of consent in Ghana, allowing for full informed participation.

*Exclusion criteria:* Individuals below 18 years of age, those not registered with any disability organisation, and individuals who were unwilling to provide informed consent were excluded from the study.

### 2.5 Sample Size

Data collection continued until theoretical saturation was reached, which occurred at the 21st participant, after whom no new themes or information emerged. The selection of 21 participants across five districts was guided by the principle of purposive sampling and theoretical saturation rather than statistical representativeness, which is consistent with qualitative phenomenological research. In qualitative research, sample size is not determined by the number of sites or the size of the population, but by the depth and richness of information obtained [17]. Each district contributed participants who brought distinct socioeconomic backgrounds and lived experiences, ensuring diversity in the data. The approach is in line with comparable phenomenological research carried out in low‐ and middle‐income countries, where it is generally acknowledged that samples of 15–25 individuals are sufficient to reach saturation [18].

### 2.6 Data Processing

Fieldwork interviews were transcribed from audio to English texts verbatim. To ensure familiarity with the data, the transcripts were read multiple times, with notes taken to facilitate the coding process.

### 2.7 Data Analysis

Data analysis followed Braun and Clarke's [19] six‐phase approach to thematic analysis. In the first phase, initial codes were systematically developed across the whole dataset, where each code was applied to a single unit of meaning that was relevant to the research objectives. The second phase involved organizing the codes into potential themes by searching for patterns and relationships within the data. The third phase involved reviewing and refining themes by checking them against the coded extracts and the entire data set to ensure the themes accurately reflected the data. In the fourth phase, themes were defined and named clearly to capture the conceptual content. In the fifth phase, the analysis was written up, and selected participant quotes were used to illustrate and confirm each theme, ensuring that the findings were grounded in the participants' own words. To improve the credibility and consistency of the analytical process, two members of the research team coded specific sections of the transcripts separately. All thematic analysis was conducted manually without the use of qualitative data analysis software. No statistical software was used, as this study employed a qualitative phenomenological design with no quantitative data analysis.

### 2.8 Data Trustworthiness

The data were credible because the researcher ensured that the data accurately reflected the perceptions that participants had and their experiences with regard to the study. This was achieved when the researcher had prolonged interview sessions with participants in which they were encouraged to give detailed answers that were honest. Additionally, triangulation was employed by engaging individuals who have different forms of disabilities from different districts, therefore increasing the validity of the study's findings.

Data privacy and protection were also ensured throughout the study. All audio recordings and transcripts were stored securely and accessed only by the research team. Participants were assigned codes (e.g., Participant 1, Participant 2) to ensure anonymity, and no personally identifiable information was included in the transcripts or final report. Data will be retained securely for a period consistent with institutional guidelines and destroyed thereafter.

### 2.9 Ethical Issues

The Kwame Nkrumah University of Science and Technology's Committee on Human Research Publication and Ethics (CHRPE) granted ethical approval (approval number: CHRPE/AP/491/25). The researcher considered other ethical issues, which included seeking verbal consent from all the participants before recording them, explaining the details and purpose of the study to the participants, and ensuring confidentiality and anonymity. To ensure voluntary involvement, the researcher made sure that only those who had completed the consent form were allowed to take part in the study.

## 3 Results

### 3.1 Demographic Characteristics

Table 1 shows that the participants were relatively young, with 47.7% between 26 and 35 years old. Males make up 57.1%, while females account for 42.9%; 61.9% had physical disabilities, and 38.1% were hearing‐impaired. In terms of education, 52.4% have attained tertiary education, with 38.1% in business/entrepreneurship, 19% in education, and 14.3% as artisans.

**Table 1:** Demographic characteristics of participants.

Variables	Frequency (F)	Percentage (%)
**Age group (years)**		
26–35	10	47.7
36–45	4	19.0
46–55	3	14.3
56–65	2	9.5
66–75	2	9.5
**Gender**		
Male	12	57.1
Female	9	42.9
**Type of disability**		
Physically challenged	13	61.9
Hearing impaired	8	38.1
**Educational level**		
Tertiary	11	52.4
Senior high school	5	23.8
Basic school	3	14.3
No formal education	2	9.5
**Job sector**		
Business/entrepreneur	8	38.1
Artisans	3	14.3
Health workers	1	4.8
Education sector	4	19.0
Local government	1	4.8
Student	2	9.5
Retired worker	2	9.5
**Total**	**21**	**100**

*Note:* Barriers to access and utilization of assistive devices.

Assistive devices can be life‐changing for persons with disabilities, but accessing and utilizing them is often hindered by several barriers such as financial constraints, lack of awareness, inadequate infrastructure, and social stigma.

### 3.2 Cost of Assistive Device

There are several reasons why assistive technology is so expensive. The dependence on imports is a major factor because in Ghana, it might not be possible to manufacture these gadgets domestically, which would raise prices because of import expenses and taxes. Additionally, these gadgets may become more expensive due to the necessity to fit and customize them for specific consumers. The high cost of these devices may also be a result of limited local production and related economies of scale, making them unaffordable for those with physical disabilities. This problem is made worse by the fact that most people with physical disabilities do not have stable employment, which leaves them with little money. As a result, individuals frequently depend on family members or caretakers to buy these gadgets for them, adding to the strain on these support networks.

### 3.3 A Participant Recalled

*“Our assistive devices are expensive because we buy them, and formally it wasn't costing too much, but these days it is very costly. I recently purchased one, and it cost me almost 2,500. As a pensioner, if I don't solicit, how can I get one? I'm a board member, so it was easy for me to get access to one, but what of those who are not board members? So it becomes a challenge”. (Participant 14, individual interview)*.

### 3.4 Another Participant Gave This Response

*“The prices of the assistive devices have become unbearable. I heard that they are expensive because the materials that are used to produce them is expensive when they ship them so when you go to places like Begoro, for them to customize an artificial leg for you is very costly and we are unable to afford.”. (Participant 2, individual interview*). These accounts reveal not only a financial inconvenience but also a systemic exclusion caused by Ghana's reliance on imported assistive devices and the absence of local manufacturing capacity. The burden is carried excessively by those with disabilities who are already economically disadvantaged, creating a compounding cycle of poverty and exclusion.

### 3.5 Centralization of Sales Points of Assistive Devices

The accessibility issues faced by individuals with physical challenges in obtaining assistive devices can be attributed to several factors. The participants talked about limited distribution channels, where these devices are often only available in specific locations, typically in urban areas, making it difficult for those in rural or hard‐to‐reach areas to access them. They added that, additionally, the lack of distribution and providers not ordering enough supplies can result in the devices being unavailable, and this further limits access. This, according to the participants, is forced to travel long distances to obtain these devices, which can be time‐consuming and costly, ultimately hindering their ability to acquire and utilize the necessary assistive technology.

*“Getting it when spoilt is very difficult. You won't be able to get some. It's not available” (*Participant 5, individual interview*)*

*“Getting the device was not easy because you had to move to Koforidua before you could get the device. Some whites would take the measurements, and when it's ready, you need to travel all the way to Koforidua for it. It was not accessible.” (*Participant 13, individual interview*)*. Participants' experiences reveal a structural inequality in the delivery of assistive technology services, where proximity to urban centres is a factor influencing access. This reflects a wider pattern of centralisation of health services in Ghana that disadvantages those in rural districts.

### 3.6 Societal Attitudes and Stigmatization Towards the Use of Assistive Devices

Another barrier that caused participants not to use an assistive device was due to stigmatization and prejudice by persons without disability. Persons with disabilities are often seen as incapable of doing anything for themselves when they use these assistive devices. They are being pitied and stigmatized. This disturbs them a lot, and most of them decide not to use the assistive device because if you do not see him or her with an assistive device, you do not get to stare at him.

*“Sometimes people will stigmatize you when you use an assistive device. They think you are incompetent and then do not want to even communicate with you once they see that you are using an assistive device”. (Participant 6, individual interview)*

*“People will look at you when you are using this device. They can stare at you, and you will become disturbed, so you might even decide not to use the assistive device anymore because it is very uncomfortable”. (Participant 10, individual interview)*. These findings show that stigma isn't just a social experience, it actively stops people from using the very devices meant to help them. When someone repeatedly faces judgment or stares, they start to get affected by those reactions, and over time, leaving the device at home to fit in becomes the next choice.

### 3.7 Inaccessible Infrastructure and Environment

People with disabilities face major obstacles due to inadequate infrastructure, which restricts their ability to participate in society. One of the main causes raised by the participants is that existing buildings are frequently created and constructed without taking accessibility into account, which reflects a lack of prioritization in the past. Another issue which the participants brought up was that there are frequently persistent gaps in the enforcement of accessibility standards in public spaces and construction rules. Efforts have been made to integrate designs that would increase inclusion into our transport systems and buildings, but this may not have been possible due to financial constraints or conflicting goals. Consequently, an inaccessible environment prevents assistive devices from being used effectively by users, and this affects their daily lives. This shows why infrastructural design must prioritize accessibility to promote equal participation.

*“As for getting a car, they won't pick you up. For them, getting down and carrying you into the car and then also putting your wheelchair in the car, they won't do that job. Even when it's raining, they will not pick you up” (Participant 5, individual interview)*

*“I once did my service as a teacher, and all the classes around were not disability friendly. Even here, where I work now, it is disability friendly, but it is very steep, and if you joke, you will roll on the ground” (Participant 17, individual interview). The experiences of the participants highlight the gap between the environment practice in Ghana and policies on disability where there are laws concerning disabilities but there is no implementation*.

### 3.8 Coping Strategies for Overcoming Barriers

Individuals with disabilities employ the use of different coping mechanisms to overcome the barriers which they face day in and day out in their lives. This section explores the various strategies as well as the roles which various institutions play in the facilitation of access, and suggestions for improvement. By examining the experiences of individuals with disabilities, we will be able to identify the ways in which individuals with disabilities overcome their barriers and gain ways to enhance accessibility of assistive technologies in our communities.

### 3.9 Adaptation Through Practice

The findings of the study showed that individuals with disabilities have adapted to the use of assistive devices through daily practice. This has been a coping mechanism for them, which they have used to overcome their difficulty in using the assistive devices. This is because the more an individual uses a device, the more he or she becomes used to the device and comfortable with the device, which allows the individual to adjust to the challenge. Through the consistent use of the device, the brain has a part that adapts to the new experience when an activity is being repeated overtime, and this helps as a coping mechanism for them. Through this adaptation process, the users can turn their device into a tool that helps them with their independence.

*“Practise makes a man perfect. So, using the gadget every day, you will become more experienced and used to it. At times I forget I'm even wearing a caliper”* (Participant 14, individual interview).

*“As for me, when you wear it, the challenges are there because you are not used to it after the operation, so if you aren't persistent, you will remove the device, but if you take it up every day, it will make you used to the device and then you will use it all the time and that is what will help you. Making up your mind to use it all the time, later you become used to it*” (Participant 7, individual interview).

### 3.10 Mental Resilience

The experiences of participants highlight the importance of the mind when it comes to the use of assistive devices. An important fact is that accepting their situation helps them to overcome this barrier of assistive device usage, allowing individuals to be determined in device usage. By making up their mind and making a conscious effort to use the device, they overcome their initial challenges. This acceptance empowers them to use their assistive device as a tool that aids independence rather than a burden to them, and it enhances their social participation.

*“As for the challenge, it is there, so what I do is that when you have money, things become easy. So, it's on you, the individual, to pray and be strong‐willed to accept your new situation and prepare mentally for it” (*Participant 5, individual interview)

*“I put it at the back of my mind that I will always focus on what I want to achieve. I was mentally ready and focused, and that I won't make anything intimidate me in using the assistive device, and that is why I possess a master's in education psychology” (Participant 10, individual interview)*

### 3.11 External Support

The experiences threw light on the important role that external support from individuals help in facilitating access to assistive devices. This is because support systems such as families, friends, and associations can reduce financial barriers that prevent access. The study found that family members help individuals with disabilities by providing financial assistance to them, and this removes cost as a barrier to the individual with disability. The associations may offer help by providing assistive devices for the individual in need, and this increases access. Furthermore, the emotional support these networks provide inspires people to look for and use assistive technology, highlighting the significance of community in improving the quality of life for individuals with disabilities.

*“I did not buy my hearing aid from Ghana. My fellow lecturers helped me to get one. I did not know how to get hold of one; they ordered it from Germany for me”* (Participant 20, individual interview)

*“\I join sports, so with the challenge of getting is not my problem, it is easy to acquire the device. When you join the sports team, they give it to you because you need one to train, and they also give you one for personal use”* (Participant 4, individual interview).

### 3.12 Comparison Across Disability Groups

Both groups faced many of the same problems and used similar ways to cope. But their biggest challenges were different. People with hearing impairments talked more about stigma and feeling left out. Hearing aids are small, so people do not see them like they see a wheelchair or crutches. But when someone notices it, they often misunderstand it or stare, which makes things uncomfortable. People with physical impairments, on the other hand, struggled more with physical barriers. Things like stairs, bad roads, and buildings without ramps made moving around hard. Money was also a big issue. Devices like prosthetics and calipers cost a lot, and replacing them when they break is not easy. When it came to coping, the two groups handled things differently too. People with physical impairments said practice and getting used to their devices helped them most. People with hearing impairments relied more on family, friends, and support groups, and many got their devices from abroad.

## 4 Discussion

The results of this study are interpreted using the International Classification of Functioning, Disability and Health (ICF) framework [20], which conceptualizes disability not as an individual deficit but as the outcome of an interaction between a person's health condition and their environmental and personal contextual factors. This theoretical view demonstrates that the barriers identified in this study high cost, limited availability, inaccessible infrastructure, and stigma are not individual experiences but are manifestations of systemic environmental barriers. The ICF framework categorizes these barriers as products of societal structures, policies, and attitudes. On the contrary, the coping strategies of adaptation through practice, mental resilience, and external support are the personal and social facilitators in the ICF model that people mobilize when there is no adequate systemic support. From this perspective, the findings are reframed from individual limitation to structural and societal responsibility with implications for policy and practice. The cost of the assistive device served as a barrier that hinders a lot of people from getting access to an assistive device. Most people with disabilities are not able to afford it since it is expensive in the country. This study had lots of participants talk about the cost of the device being a challenge to them whenever they want to purchase an assistive device. This confirms a study by Boot et al. [21] where a global systematic review was conducted on assistive technology, where the analysis of 22 studies revealed 77 barriers overall, with high cost as one of the most frequently reported obstacles. This aligns with a study that talks about hearing aids typically costing around GH₵10,000 (USD 990), against a national daily minimum wage of GH₵13.5 (USD 1), and as a result, this affects persons with hearing impairment from being able to afford these assistive devices, which limits their access to assistive hearing technology and amplifies socioeconomic exclusion [22]. The implication of this is that the high cost of assistive devices in Ghana limits access for people with disabilities, exacerbating socioeconomic exclusion. When these devices are very expensive for those who really need them, it becomes a major barrier. It simply means people who are in low‐income situations will not be able to get the help they need. This raises concern for the need for interventions to improve affordability, and such interventions could be reductions in the costs of assistive devices, or manufacturing initiatives which would make the devices more accessible.

Another challenge that came up during the data collection was that places where assistive devices were available were very limited. They can be accessed at particular places. Participants identified the distance as a barrier, and assistive devices were very scarce. This aligns with a study by Tangcharoensathien et al. [23], which highlights that assistive technologies are able to enhance the lives of persons with disabilities; however, they are not available or are too expensive in many areas. Assistive devices being limited and not accessible affect those in underserved areas, therefore, causing inequalities in healthcare and also preventing social participation. Assistive devices being limited and expensive have a very significant implication on persons who need them, as this leads to the prevention of social participation, and in the long run limits the opportunities for social interaction and education. This issue can be addressed when the government increases investments, provides a strong distribution network, and integrates assistive technology into our health systems to ensure there is equal access [24].

The problem of stigmatization was another significant influence that surfaced. According to Lynch et al. [25], the social experiences that individuals with disabilities have had in regard to using assistive devices may discourage them from using them. The participants reported experiencing stigmatization occasionally due to the usage of assistive devices, such as wheelchairs and hearing aids. This occasionally made them desire to withdraw socially and avoid going out to prevent being viewed negatively. This implies that stigmatization associated with assistive device use leads to social withdrawal and reduced participation, further marginalizing people with disabilities and hindering their integration into society. This is confirmed in a study by Tsatsou [26], which concludes that disability stigma suggests that the experiences that persons with disabilities have around technology can affect their social participation and sense of inclusion, and it is supported by the views that participants expressed in the study. It is also important to note that the barriers and coping strategies experienced were not similar across the two disability groups studied. Persons with hearing impairments encountered unique social challenges caused by the visibility and stigma of hearing aids, whereas persons with physical impairments were more directly impacted by infrastructure gaps and the exorbitant cost of mobility aids. This supports the claim made by Visagie et al. [7] that disability‐specific interventions are required because a single policy approach might not be sufficient to meet the diverse needs of people with various disabilities.

The results of the study reflect how individuals with hearing and physical impairment have developed both personal and communal ways to navigate these challenges. Most of the participants became used to the use of their assistive device. The assistive device has become a part of them, and they are now used to it. This is evident in a report by Robertson [27], which explored the acceptance of prosthetic and embodiment among individuals with upper limb impairment, made emphasis that using the devices for a while contributed to the integration into the users' experience. Some felt pains while using the device initially but, using the device constantly has made them used to the device now that they feel the device is part of their body, so they do not feel the pain anymore and a similar response can be found in a study by Joskow et al. [28] that the constant usage of assistive device has shown to improve adaptation among persons with disabilities. Another coping strategy the participants use is having mental resilience. The participants motivate themselves and accept their new selves. They embrace their new identity, which helps them accept who they are and acknowledge the use of an assistive device. A study which was conducted in Nigeria, the Oyo State, revealed the positive attitudes of hearing‐impaired students towards the use of assistive technology in the classroom after they were willing to adopt and use the device [29]. This suggests that through adaptation and mental resilience, individuals with disabilities can integrate assistive devices into their lives, enhancing participation and quality of life.

Financial constraints served as a barrier to the use of the assistive device. Most of the participants found the assistive device to be expensive. Monden et al. [30] reported similar findings where financial constraint was identified as a significant barrier that affected the use of assistive devices, as the cost of purchasing the assistive technology limited their access. The participants talked about external support helping them to overcome these challenges. External support will help those that are not able to purchase the device get some of the device as news report from [31] outlines that with Zambian government's proactive effort to improve access to assistive devices, there was a collaboration between the Zambia Agency for Persons with Disabilities, the church of Latter‐day Saints and the Ministry of Community Development and Social Services provided over 450 assistive devices to persons with disabilities. Such collaboration addresses challenges in financial constraints and affordability. This highlights how collaboration between government, associations, and even institutions can help address financial barriers and improve access for those in need of the device.

### 4.1 Limitation

One limitation of the study is its focus on specific districts in Ghana's Ashanti Region, which gives strong emphasis on transferability and might limit generalizability to other countries. The qualitative approach, on the other hand, while providing in‐depth information, may not be representative of the broader population. The study's goal was to investigate barriers and adaptations in a particular setting, offering important insights into the experiences of people with disabilities in this area; hence, the restrictions had no effect on the findings. The application of thematic analysis and data triangulation improved the study's reliability and validity.

## 5 Conclusion

The study makes the following specific policy and practice recommendations to address the identified barriers. First, the Government of Ghana through the Ministry of Health and the National Council on Persons with Disabilities should establish a special assistive technology subsidy fund for low‐income persons with disabilities. The eligibility criteria should be tested and based on disability certification. Secondly, the Ministry of Health should collaborate with the district health directorates to decentralize the distribution of assistive devices by establishing fitting and maintenance centers in each district. This will reduce the need for persons with disabilities to travel to urban centers such as Koforidua. Thirdly, the Ghana Education Service and the Commission on Human Rights and Administrative Justice (CHRAJ) should develop and implement mandatory disability‐inclusive infrastructure standards for all public buildings, with a specific timeline and accountability mechanism for retrofitting existing structures under the Persons with Disability Act, 2006 (Act 715). Fourthly, the National Commission for Civic Education (NCCE) should partner with disability advocacy organizations to develop and implement community‐based stigma reduction campaigns based on the lived experiences of persons with disabilities. This would involve moving beyond broad awareness messaging to targeted, culturally sensitive discussion at the community level. Fifth, future research should consider the separate experiences of persons with physical and hearing disabilities and the role of digital assistive technologies in bridging access gaps in the Ghanaian context.

## Author Contributions

**Jeffrey Kwame Owusu Ansah:** writing – original draft, conceptualization, investigation, methodology, data curation, and resources. **Enoch Acheampong:** supervision, writing – review and editing, and conceptualization. **Ros Yaw Owusu‐Ansah:** data curation, methodology and writing – original draft. **Grace Kisiwaa Agyei:** methodology, data curation and investigation. **Vida Kasore:** supervision, writing – editing and review, and methodology. **Justice Owusu Agyemang:** conceptualization and writing – original draft. **Betty Kponyo:** data curation and validation. **Elizabeth Owusu Ansah:** methodology, writing – review and editing, and data curation. **Jerry John Kponyo:** supervision, conceptualization, and resources.

## Funding

The authors have nothing to report.

## Disclosure

All authors have read and approved the final version of the manuscript. Jeffrey Kwame Owusu Ansah had full access to all of the data in this study and takes complete responsibility for the integrity of the data and the accuracy of the data analysis.

## Conflicts of Interest

The authors declare no conflicts of interest.

## Transparency Statement

The lead author, Jeffrey Kwame Owusu Ansah, affirms that this manuscript is an honest, accurate, and transparent account of the study being reported; that no important aspects of the study have been omitted; and that any discrepancies from the study have been explained.

## Data Availability

The data sets used and analyzed during the current study are available from the corresponding author upon reasonable request.
